# Blocked versus randomized presentation modes differentially modulate feedback-related negativity and P3b amplitudes

**DOI:** 10.1016/j.clinph.2013.09.029

**Published:** 2014-04

**Authors:** Daniela M. Pfabigan, Michael Zeiler, Claus Lamm, Uta Sailer

**Affiliations:** a Social, Cognitive and Affective Neuroscience Unit, Department of Basic Psychological Research and Research Methods, Faculty of Psychology, University of Vienna, Liebiggasse 5, A-1010 Vienna, Austria; b Department of Psychology, Faculty of Social Sciences, University of Gothenburg, Haraldsgatan 1, SE-40530 Gothenburg, Sweden

**Keywords:** FRN, P3b, Feedback stimulus characteristics, Presentation mode

## Abstract

**Objective:**

Electrophysiological studies on feedback processing typically use a wide range of feedback stimuli which might not always be comparable. The current study investigated whether two indicators of feedback processing – feedback-related negativity (FRN) and P3b – differ for feedback stimuli with explicit (facial expressions) or assigned valence information (symbols). In addition, we assessed whether presenting feedback in either a trial-by-trial or a block-wise fashion affected these ERPs.

**Methods:**

EEG was recorded in three experiments while participants performed a time estimation task and received two different types of performance feedback.

**Results:**

Only P3b amplitudes varied consistently in response to feedback type for both presentation types. Moreover, the blocked feedback type presentation yielded more distinct FRN peaks, higher effect sizes, and a significant relation between FRN amplitudes and behavioral task performance measures.

**Conclusion:**

Both stimulus type and presentation mode may provoke systematic changes in feedback-related ERPs. The current findings point at important potential confounds that need to be controlled for when designing FRN or P3b studies.

**Significance:**

Studies investigating P3b amplitudes using mixed types of stimuli have to be interpreted with caution. Furthermore, we suggest implementing a blocked presentation format when presenting different feedback types within the same experiment.

## Highlights

• ERP responses to feedback stimuli with explicit or assigned valence information were investigated with blocked or randomized trial presentation modes.
• Only P3b, but not feedback-related negativity amplitudes were affected by feedback type for both presentation modes.
• Results suggest using blocked design when using different types of feedback stimuli.

## 1 Introduction

The monitoring of ongoing events – whether they concern internal states or external affairs – is crucial in daily life. Human neuroscience research has addressed internal and external performance monitoring extensively for over two decades now. Much of this research has relied on the use of event-related potentials (ERPs) which allow investigating the neuronal correlates of performance monitoring with a temporal resolution in the millisecond range. The feedback-related negativity (FRN; Miltner et al., 1997), which is a negative-going component peaking around 200–300 ms after the presentation of external feedback, is an ERP component that has been repeatedly used to investigate performance monitoring based on external feedback. Enhanced FRN amplitudes have been reported after negative performance feedback (Miltner et al., 1997, Nieuwenhuis et al., 2004), after unexpected events (Hajcak et al., 2007, Pfabigan et al., 2011b), after monetary losses (Gehring and Willoughby, 2002), and after salient compared to insignificant outcomes (Gehring and Willoughby, 2002, Yeung et al., 2005). The P3b (Polich, 2007) is another ERP component repeatedly observed in situations requiring performance monitoring. It is a positive-going component peaking in the time window of 300–600 ms after external feedback presentation. P3b amplitude variation has been found to be sensitive to stimulus significance, the probability of occurrence of a stimulus (Duncan Johnson and Donchin, 1977), as well as to task and stimulus complexity (Isreal et al., 1980, Johnson, 1986) and effort spent on a task (Brocke et al., 1997).

More than one hundred studies on ERP correlates of feedback processing have been published so far, and there is considerable variation of the feedback stimuli used in these studies. Notably, feedback stimuli might differ with respect to a variety of factors, such as how much perceptual or cognitive processing they require (Zhang et al., 2012). For example, simple symbols like x, o, +, − have often been presented to indicate feedback valence via prior assignment (Hajcak et al., 2006, Hajcak et al., 2007, Holroyd et al., 2006, Miltner et al., 1997, Sato et al., 2005), while numbers (sometimes in different colors) as well as pictures of coins served as feedback stimuli to indicate the amount of monetary gain or loss more indirectly (Bellebaum and Daum, 2008, Bellebaum et al., 2010, Donamayor et al., 2012, Gehring and Willoughby, 2002, Pfabigan et al., 2011a, Sailer et al., 2010, Wu and Zhou, 2009, Yeung and Sanfey, 2004, Yu and Zhou, 2006). In addition, some studies have used social stimuli such as faces with either neutral (Warren and Holroyd, 2012, Zhang et al., 2012) or emotional facial expressions (such as anger, sadness or happiness; (Li et al., 2011, Pfabigan et al., 2011a, Schulreich et al., 2013) to explicitly indicate feedback valence. Feedback stimuli such as faces depicting basic facial emotion expressions contain valence information that can be recognized directly and universally (Ekman and Friesen, 1976). Furthermore, emotional facial expressions are considered to be important social cues comprising necessary information in social exchange situations (Rolls, 2000), conveying feedback valence without prior learning requirements (van der Veen et al., 2011). In contrast, the valence assignment for + and − symbols has to be learned before being used as valence indicator. Consequently, the question arises whether explicit or assigned valence information results in different feedback processing.

Notably, answering this question requires experimental designs that compare feedback-related neural signals within the same individuals – as individual variation across subjects might prevent the detection of potentially unique differences in feedback processing when relying on a between-subject design. As of yet, only one cognitive neuroscience study addressed the question whether different types of feedback stimuli influence neuronal activity during feedback processing within the same individuals. Using functional magnetic resonance imaging (fMRI), van der Veen et al. (2011) administered a time estimation task (Miltner et al., 1997) and used either emotional faces or verbal statements as feedback stimuli – with feedback type being randomly varied across the experiment. Their hypothesis was that facial feedback contained more direct emotional value and would lead to enhanced neuronal activation in brain areas associated with feedback processing, in comparison to verbal feedback. Although it was observed that facial feedback activated a generally larger neuronal network than verbal feedback with higher activation in occipital areas and the left inferior temporal gyrus, neuronal activation was comparable in brain areas associated with feedback processing when contrasting the two different feedback types. However, the poor temporal resolution of fMRI might not have permitted an adequate detection of the rapid neuronal changes known to be associated with feedback processing. Furthermore, electrophysiological indices of brain activity might provide access to aspects of neural processing that remain undetected by hemodynamic activation measures.

Thus, the current study is the first to apply a within-subject design to directly compare ERP indicators of feedback processing using feedback stimuli with explicit or assigned valence information. In principle, stimuli with assigned valence information (such as + and −) contain valence information comparable to stimuli with explicit information (such as emotional facial expression). However, we were interested in whether emotional facial expressions added additional saliency to the feedback stimuli which might be reflected in enhanced ERP amplitudes. Our assumption of emotions impacting FRN amplitude variation is further supported by the observation that even slightly elevated levels of self-reported state and trait negative affect are associated with FRN enhancement after negative feedback (Santesso et al., 2012). Moreover, Santesso et al. (2012) suggested that FRN amplitude variation might be context-dependent, with negatively-valenced contexts eliciting larger FRN amplitudes. Thus, the question arises whether or not explicit negative feedback stimuli (i.e., angry facial expressions) have a comparable context effect on FRN amplitudes.

Concerning the P3b component, amplitude variation has been reported in response to negatively- as well as in response to positively-valenced stimuli. However, the picture is far from consistent. Larger P3b amplitudes after positive feedback have been reported several times (Bellebaum et al., 2010, Hajcak et al., 2007, Pfabigan et al., 2011b). Other studies reported no differences in P3b amplitude variation for positive and negative outcomes (Sato et al., 2005, Yeung and Sanfey, 2004). Schuermann et al. (2012) and Frank et al. (2005) found P3b amplitude enhancement after the presentation of negatively valenced stimuli. Research on emotional picture content suggested that P3b amplitude enhancement is elicited by the presentation of emotionally charged pictures in comparison to neutral pictures (Briggs and Martin, 2009, Keil et al., 2002). Based on this assumption, Yeung et al. (2005) suggested that P3b enhancement during feedback processing might reflect higher subjective task involvement. More precisely, P3b enhancement might also reflect the affective significance of the presented feedback stimuli (Yeung et al., 2005).

We conducted three separate experiments investigating the impact of different feedback stimuli – explicit versus assigned stimuli (i.e., emotional facial expressions versus symbols) – on neuronal correlates of feedback processing. We expected larger FRN amplitudes after negative than after positive feedback (Miltner et al., 1997), particularly for explicit stimuli (Santesso et al., 2012). The explicit stimuli were social stimuli which are thought to be crucial for behavioral adaptations (Rolls, 2000). On a longer time scale, recognizing emotional facial expressions has been proposed to be evolutionarily adaptive because it facilitates social interaction, helps to avoid threats and thereby enhances an individual’s likelihood of survival (Vaish et al., 2008).

Concerning later stages of feedback processing, we expected larger P3b amplitudes after positive than after negative feedback stimuli (Bellebaum et al., 2010, Pfabigan et al., 2011b). In particular, we expected larger P3b amplitudes for explicit than for assigned feedback because of higher salience (Yeung and Sanfey, 2004) and higher stimulus complexity (Isreal et al., 1980, Johnson, 1986). Furthermore, we explored behavioral measures of time estimation and their relation to FRN and P3b amplitudes variation. Empirical evidence suggests that the larger FRN amplitudes, the larger the corresponding behavioral modifications (Holroyd and Krigolson, 2007, van der Helden et al., 2010). This is in line with the assumption that the anterior midcingulate cortex (aMCC), the most likely source of the FRN component (Gehring and Willoughby, 2002, Holroyd and Coles, 2002, Miltner et al., 1997), is implicated in behavioral adaptation (Holroyd and Coles, 2002).

In addition to investigating explicit versus assigned feedback stimuli, two further experiments were performed to corroborate the results from experiment 1 and to explore the effect of different presentation modes on FRN and P3b amplitudes. In the literature, mixed (i.e., randomized) and blocked presentation modes are typically assumed to be comparable and alternative designs. However, since mixed versus blocked presentation may potentially influence subjective stimulus predictability, which in turn has been found to affect the FRN and the P3b, these different modes may per se influence ERP amplitudes.

## 2 Experiment 1

Experiment 1 comprised of a time estimation paradigm where the explicit and assigned feedback stimuli were presented randomly mixed.

### 2.1 Methods

#### 2.1.1 Participants

Initially, 20 volunteers (eleven females) participated in experiment 1. Two participants (one female, one male) had to be excluded from further analysis due to EEG data acquisition problems. The remaining 18 participants were aged between 21 and 35 years, with a mean age of 26 ± 4.01 years. For behavioral data analysis, only 16 datasets (nine female) were available due to technical problems. Participants were right-handed as assessed with the Edinburgh Handedness Inventory (Oldfield, 1971), had normal or corrected-to-normal vision, and reported no past or present neurological or psychiatric disorder. The current study was conducted in accordance with the Declaration of Helsinki (revised 2000) and local guidelines of the University of Vienna. Participants were required to give written informed consent prior to the experiment. According to the Austrian Universities Act 2002 (UG 2002) which held at the time the study was conducted, no formal approval of an ethics committee was required. However, the experiment was supervised and ethically approved by the head of the former Brain Research Laboratory of the Faculty of Psychology, University of Vienna, to guarantee high international ethical standards.

#### 2.1.2 Task

Stimulus presentation and synchronization (Pentium IV, 3.00 GHz) with the electroencephalogram (EEG) recordings was controlled by E-Prime 2.0 software (Psychology Software Tools, Inc., Sharpsburg, PA). For EEG data collection, participants were seated comfortably in a sound-attenuated room about 70 cm in front of a 21” cathode ray tube monitor (Sony GDM-F520; 75 Hz refresh rate). A modified version of the time estimation task by Miltner et al. (1997) was used as the experimental paradigm. Participants’ task was to estimate the duration of one second and indicate their estimation via button press. Each trial started with the presentation of a black fixation dot presented centrally on a gray screen. After 1000 ms a black star replaced the dot for 250 ms. The star indicated the starting point of each time estimation. Subsequently, a blank gray screen was presented for 1750 ms. During this period, participants could indicate the estimated elapse of one second via pressing button 1 on a response pad, using the index finger of their dominant hand. Exactly 2000 ms after the onset of the time estimation, feedback was presented for 1000 ms to indicate the accuracy of time estimation. The subsequent inter-trial-interval depicted again the black fixation dot and varied randomly between 1400 and 1600 ms. Feedback was provided based on individual performance. However, task difficulty was adjusted to the individual performance level to guarantee comparable numbers of correct and incorrect trials. Each participant started initially with the following criteria: Positive feedback was given in cases where the button press fell in the time window of 900–1100 ms after the onset of the star. Subsequently, the width of this time window was automatically adjusted based on individual performance on the preceding trial (Miltner et al., 1997). After a trial with positive feedback (i.e., a correct time estimation), the time window was narrowed down by 10 ms at both ends of the window (e.g., 910–1090 ms after the initial trial). After a trial with negative feedback (i.e., an incorrect time estimation), the time window became widened again by adding 10 ms at both ends. Consequently, the overall probability of positive and negative feedback was approximately 50%. All feedback stimuli were equiluminescent and comparable in size (4 × 5 cm). Two different types of feedback stimuli were used – emotional facial expressions and symbols as explicit and assigned feedback cues, respectively. The assigned cues consisted of “+” (indicating positive feedback) and “−” (indicating negative feedback) signs. The explicit cues consisted of photographs of one male poser of the Pictures of Facial Affect database (Ekman and Friesen, 1976). The happy facial expression indicated positive feedback; the angry facial expression indicated negative feedback. Happy and angry faces were chosen to represent positive and negative feedback because the valence difference between these two facial expressions is known to be maximal (Russell and Bullock, 1985). Participants were instructed in detail that a “+” symbol and the happy face both indicated correct time estimation and that the “−” symbol and the angry face both indicated incorrect time estimation. Thus, symbols and faces were equivalent indicators of correctness of time estimation. The experiment consisted of 20 training trials and 400 experimental trials. The 200 trials depicting explicit feedback stimuli and the 200 trials depicting assigned feedback stimuli were presented randomly intermixed. The experimental trials were divided into eight blocks of 50 trials each to offer participants short rests during the experiment. Overall EEG data collection lasted around 40 min.

#### 2.1.3 EEG data acquisition

EEG was recorded via six Ag/AgCl ring electrodes embedded in a fabric electrode cap (EASYCAP GmbH, Herrsching, Germany; model M10) from the mid-line electrode locations Fz, FCz, Cz, PCz, Pz, and Oz. The current experiment used this reduced electrode setting because FRN and P3b amplitudes are typically measured from midline electrode locations. Additionally, four electrodes were placed 1 cm above and below the left eye, and on the outer canthi to measure horizontal and vertical electro-oculogram (EOG) via a bipolar setting. These EOG signals were used off-line for eye movement correction. Two additional electrodes were placed above the seventh vertebra and on the right sterno-clavicular joint to serve as reference sites for EEG recording (Stephenson and Gibbs, 1951). Electrode impedance was kept below 2 kΩ via a skin-scratching procedure applied at each electrode site (Picton and Hillyard, 1972) and the insertion of degassed electrode gel (Electrode-Cap International, Inc., Eaton, OH). Signals were amplified using an AC amplifier set-up with a time constant of 10 s (Ing. Kurt Zickler GmbH, Pfaffstätten, Austria), and sampled at 250 Hz for digital storage.

#### 2.1.4 Behavioral data analysis

For descriptive analysis, the percentage of positive and negative feedback conditions was calculated across all participants. Additionally, mean response times were calculated across all participants and trials to describe whether the one second interval was over- or underestimated in general. Differences in response times were calculated per participant between each trial and its preceding trial separately for positive and negative feedback to describe changes in response times evoked by directly preceding feedback more precisely. These trial-to-trial changes in response time were subjected to a 2 × 2 repeated-measures ANOVA with the within-subject factors *feedback type* (explicit, assigned) and *valence* (negative, positive) to investigate time estimation changes as a function of feedback type. Furthermore, it was assessed whether the different feedback types led to more appropriate adjustments in time estimation. The relative frequencies of correct and incorrect time adjustments were calculated subject-wise and submitted to the same 2 × 2 repeated-measures ANOVA model with the within-subject factors *feedback type* and *valence*.

#### 2.1.5 EEG data analysis

Prior to data analysis, participant- and channel-specific weighting coefficients were calculated for vertical and horizontal eye movements which were assessed during two pre-experimental calibration trials. Subsequently, these weighted EOG signals were subtracted from experimental EEG data (Bauer and Lauber, 1979). Off-line data analysis was carried out using EEGLAB 6.0.3b (Delorme and Makeig, 2004) with Matlab 7.9.0 (The MathWorks, Inc., Natick, MA). A low-pass filter with a cut-off frequency of 30 Hz (roll-off 6 dB/octave) was applied to the data. EEG data were epoched starting 200 ms prior to feedback onset and lasting 1200 ms for ERP analysis. The mean of the first 200 ms served as baseline interval. Four experimental conditions (approximately 100 trials each) were derived: *explicit negative*, *explicit positive*, *assigned negative* and *assigned positive*. A semi-automatic artifact removal procedure was applied to these epochs. Artifact-afflicted trials with voltage values exceeding ±75 μV or with voltage drifts of more than 50 μV were automatically marked by EEGLAB. During subsequent visual inspection, the automatic markings were controlled and artifact-afflicted trials were discarded from further analysis. Extended infomax independent component analysis (ICA; Bell and Sejnowski, 1995, Lee et al., 1999) was applied to the data of nine participants to remove residual eye movement-related activity which had not been removed by prior correction methods (Delorme et al., 2007). As a result of these preprocessing procedures, a minimum of two-thirds of the trials were available for further analysis in each participant. Artifact-free trials were averaged per participant and per condition. Afterwards, FRN mean amplitudes were computed condition- and subject-wise 200–300 ms after feedback onset at electrode sites Fz where FRN amplitudes were most prominent. P3b mean amplitudes were computed 300–500 ms after feedback onset at electrode site Pz.

FRN mean amplitudes were analyzed using a 2 × 2 repeated-measures ANOVA with the within-subject factors *feedback type* (explicit, assigned) and *valence* (negative, positive). P3b mean amplitudes were analyzed using the same ANOVA model. Significant interaction effects were explored with HSD Tukey post hoc tests. Furthermore, Pearson’s correlations were calculated to explore the relation between FRN (at Fz) and P3b (at Pz) amplitude variations and trial-to-trial changes in reaction time. The significance level was set at *p* < 0.05 for all statistical tests. Partial eta-squared ($\eta_{p}^{2}$) is reported to indicate effect sizes for significant ANOVA results. Values of $\eta_{p}^{2}$ = 0.01, $\eta_{p}^{2}$ = 0.06, and $\eta_{p}^{2}$ = 0.14 represent small, medium, and large effects (Kirk, 1996). Statistical analyses were performed using PASW 18 (SPSS Inc., IBM Corporation, NY) and Statistica 6.0 (StatSoft Inc., Tulsa, OK).

### 2.2 Results

#### 2.2.1 Behavioral results

The four different feedback conditions were distributed evenly across participants (*explicit negative*: 24.0%, *explicit positive*: 26.0%, *assigned negative*: 25.2%, *assigned positive*: 24.8%). Thus, participants received negative feedback in 49.2% of the 400 trials. In general, participants slightly underestimated the one-second interval (mean response time for time estimation was 967 ms ±279). Concerning trial-to-trial changes in time estimation, a main effect of *valence* was observed (*F*(1,15) = 20.02, *p* < 0.001, $\eta_{p}^{2}$ = 0.57). Trial-wise adjustments in time estimation (i.e., reaction time) were larger following negative than positive feedback for all trials. No significant effects were observed for the factor *feedback type* (*F*(1,15) = 0.82, *p* = 0.379) or the interaction (*F*(1,15) = 0.89, *p* = 0.359). Furthermore, participants adequately adjusted their time estimation (towards 1000 ms) in 76.6% of the trials after negative feedback and in 51.3% after positive feedback. Concerning the number of these correct adjustments, a main effect of *valence* was observed (*F*(1,15) = 70.39, *p* < 0.001, $\eta_{p}^{2}$ = 0.82), indicating more correct adjustments after negative than after positive feedback. Again, no significant effects emerged for the factor *feedback type* (*F*(1,15) = 0.02, *p* = 0.896) or the interaction (*F*(1,15) = 0.13, *p* = 0.722).

Trial-to-trial changes in response time and number of correct adjustments are depicted in Table 1, mean FRN and P3b amplitudes in Table 2.

**Table 1:** Mean trial-to-trial change in response time and number of correct adjustments and corresponding standard deviations (SD) of experiment 1.

Condition	Trial-to-trial change in response time		Number of correct adjustments	
	M	SD	M	SD
Explicit negative	190.34	79.75	69.75	19.18
Assigned negative	199.22	95.35	69.13	21.69
Explicit positive	130.44	31.21	44.00	15.88
Assigned positive	129.95	43.12	45.06	17.35

**Table 2:** Mean amplitude values and corresponding standard deviations (SD) of FRN at Fz and P300 at Pz in experiment 1 (*n* = 18).

		Condition			
		Explicit negative	Assigned negative	Explicit positive	Assigned positive
FRN (Fz)	Mean amplitudes	6.82	5.58	10.47	8.43
	SD	5.56	4.88	6.30	7.04

P300 (Pz)	Mean amplitudes	16.68	14.10	21.43	16.47
	SD	5.41	5.40	7.10	6.10

#### 2.2.2 EEG results

**Fig. 1:**
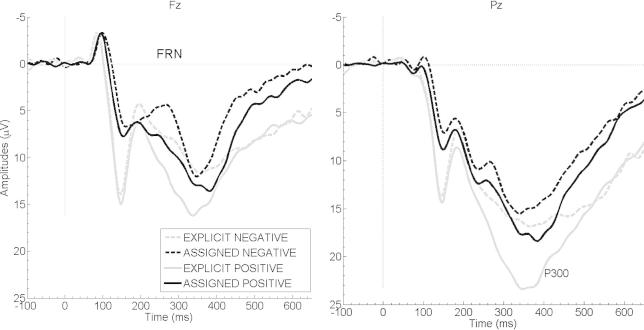
Grand average ERPs of experiment 1. Grand averages of the four conditions at electrode sites Fz (left) and Pz (right). Negative values are plotted upwards. Feedback presentation started at 0 ms, and lasted for 1000 ms.

Fig. 1 displays FRN and P3b amplitude courses of the four conditions of experiment 1. Analysis of FRN mean amplitudes revealed main effects for *feedback type* (*F*(1,17) = 6.00, *p* = 0.025, $\eta_{p}^{2}$ = 0.26) and *valence* (*F*(1,17) = 24.55, *p* < 0.001, $\eta_{p}^{2}$ = 0.59). No *feedback type* × *valence* interaction was observed (*F*(1,17) = 0.91, *p* = 0.354). FRN amplitudes were more pronounced (i.e., more negative) after negative than positive, and after assigned than explicit feedback. No significant correlations emerged between the trial-to-trial changes in response time after negative and positive feedback and FRN amplitude values (all *p*’s > 0.815).

Analysis of P3b mean amplitudes revealed main effects for the factors *feedback type* (*F*(1,17) = 20.90, *p* < 0.001, $\eta_{p}^{2}$ = 0.55) and *valence* (*F*(1,17) = 24.78, *p* < 0.001, $\eta_{p}^{2}$ = 0.59), and a significant interaction between these two (*F*(1,17) = 7.97, *p* = 0.012, $\eta_{p}^{2}$ = 0.32). Tukey post hoc test indicated that *explicit positive* feedback elicited the largest P3b amplitudes (all *p*’s < 0.001) and *assigned negative* the smallest (i.e., least positive) ones (all *p*’s < 0.005). P3b amplitudes were larger after positive than negative feedback for explicit (*p* < 0.001) and assigned feedback stimuli (*p* = 0.005); and larger after explicit than assigned feedback for positive (*p* < 0.001) and negative feedback stimuli (*p* = 0.002). *Explicit negative* and *assigned positive* feedback elicited comparable P3b amplitudes in between (*p* = 0.983). No significant correlations emerged between the trial-to-trial changes in response time after negative or positive feedback and P3b amplitude values (all *p*’s > 0.249).

### 2.3 Discussion

As expected, the FRN was larger after negative than positive feedback (Miltner et al., 1997). Negative feedback of both feedback types served as valuable information for behavioral adjustments, as indicated by increases in response accuracy following trials with negative feedback. However, no correlation of these behavioral adjustments or other behavioral measures with FRN amplitude was observed. Feedback type also impacted FRN amplitudes, however, in a rather unexpected direction since the presumably less salient assigned feedback stimuli led to more negative FRN amplitudes.

The modulation of ERPs by feedback type and valence was different for FRN and P3b components. Contrary to FRN variation, P3b analysis revealed generally higher neural responses after positive and after explicit feedback stimuli as well as an interaction effect. These results do not seem to be in line with the feedback saliency account since positive feedback is generally not assumed to be more salient (Baumeister et al., 2001). Furthermore, increased salience should also increase FRN amplitudes which was not the case for the explicit stimuli.

We therefore propose a different interpretation for our findings. In the present experiment, whether feedback was positive or negative (i.e., feedback valance), or whether feedback consisted of symbols or emotional faces (i.e., feedback type), had a probability of 50%. Feedback valence could be used to adjust performance, whereas feedback type had no behavioral implications at all. Thus, feedback type was not predictable, and this might have affected stimulus processing.

To address the possible effect of unpredictability of feedback type, we therefore conducted experiment 2 applying the same experimental paradigm with one important change. In this experiment, explicit and assigned feedback stimuli were not presented randomly mixed as in experiment 1, but in a blocked fashion. Thus, experiment 2 allowed investigating the effects of feedback type on feedback processing in a block paradigm in which the predictability of feedback type was held constant.

## 3 Experiment 2

Experiment 2 comprised of a time estimation paradigm where the explicit and assigned feedback stimuli were presented block-wise. To avoid repetition and learning effects, new participants were recruited for the second experiment.

### 3.1 Methods

#### 3.1.1 Participants

Initially, 17 volunteers (eleven females) participated in our second experiment. One female participant had to be excluded from further analysis due to technical problems. The remaining 16 participants were aged between 20 and 48 years, with a mean age of 26 ± 6.62 years. Participants were right-handed as assessed with the Edinburgh Handedness Inventory (Oldfield, 1971), had normal or corrected-to-normal vision, and reported no past or present neurological or psychiatric disorder. Thus, final sample size, age, and gender proportions of experiments 1 and 2 were very similar, maximizing comparability of the results. All participants gave written informed consent prior to the experiment. The same ethical standards applied as in experiment 1.

#### 3.1.2 Task

Experimental procedures were the same as in experiment 1 with one exception. Instead of presenting explicit and assigned feedback stimuli intermixed and randomly, blocks of the same feedback type were introduced. Each block consisted of 50 trials where only symbols or faces indicated time estimation accuracy. In total, the experiment consisted of eight blocks (400 trials). Feedback type alternated from block to block and participants were informed about this beforehand. It was randomly determined whether the first block for each participant consisted of explicit or assigned feedback stimuli. Thus, participants always knew which feedback type to expect after the first experimental trial.

#### 3.1.3 EEG data acquisition

Data acquisition and preprocessing procedures were identical to experiment 1. Data were recorded from six Ag/AgCl ring electrodes (Fz, FCz, Cz, CPz, Pz, and Oz), and the same algorithms to correct for eye movement were applied.

#### 3.1.4 Data analysis

Behavioral and EEG data analyses were identical to experiment 1. ICA was applied to the data of four participants to remove residual eye movement-related activity (Delorme et al., 2007). Subject- and condition-wise averages were calculated for the four conditions *explicit negative*, *explicit positive*, *assigned negative*, and *assigned positive*. Subsequently, FRN (200–300 ms after feedback onset) and P3b mean amplitudes (300–500 ms after feedback onset) were extracted.

### 3.2 Results

#### 3.2.1 Behavioral results

The four different feedback conditions were again distributed evenly across participants (*explicit negative*: 25.3%, *explicit positive*: 24.7%, *assigned negative*: 25.4%, *assigned positive*: 24.6%). Participants received negative feedback in 50.7% of all trials. In general, participants slightly overestimated the one second interval (mean response time for time estimation was 1021 ms ±266). Concerning trial-to-trial changes in reaction times, a main effect of *feedback valence* emerged (*F*(1,15) = 56.81, *p* < 0.001, $\eta_{p}^{2}$ = 0.79), indicating larger trial-to-trial changes in time estimation after negative than after positive feedback. The main effect of *feedback type* (*F*(1,15) = 0.30, *p* = 0.593) and the interaction (*F*(1,15) < 0.01, *p* = 0.99) did not reach significance. Participants adequately adjusted their time estimation (towards 1000 ms) in 77.4% after negative feedback trials and in 53.5% after positive feedback trials. Concerning these correct adjustments, again only a main effect of *valence* emerged (*F*(1,15) = 94.92, *p* < 0.001, $\eta_{p}^{2}$ = 0.86), indicating more accurate time estimations after negative feedback. No significant effects were observed for *feedback type* (*F*(1,15) = 0.06, *p* = 0.807) and the interaction (*F*(1,15) = 0.24, *p* = 0.63).

Trial-to-trial changes in response time, number of correct adjustments are depicted in Table 3, mean FRN and P3b amplitudes in Table 4.

**Table 3:** Mean trial-to-trial change in response time and number of correct adjustments and corresponding standard deviations (SD) of experiment 2.

Condition	Trial-to-trial change in response time		Number of correct adjustments	
	M	SD	M	SD
Explicit negative	206.84	72.15	76.47	4.76
Assigned negative	202.84	71.58	76.71	4.59
Explicit positive	142.03	53.68	52.88	12.55
Assigned positive	137.50	43.24	52.06	8.20

**Table 4:** Mean amplitude values and corresponding standard deviations (SD) of FRN at Fz and P300 at Pz in experiment 2 (*n* = 16).

		Condition			
		Explicit negative	Assigned negative	Explicit positive	Assigned positive
FRN (Fz)	Mean amplitudes	5.32	5.66	8.52	8.63
	SD	3.70	3.89	4.20	4.58

P300 (Pz)	Mean amplitudes	12.54	10.45	14.51	12.13
	SD	5.37	5.97	5.53	5.51

#### 3.2.2 EEG results

**Fig. 2:**
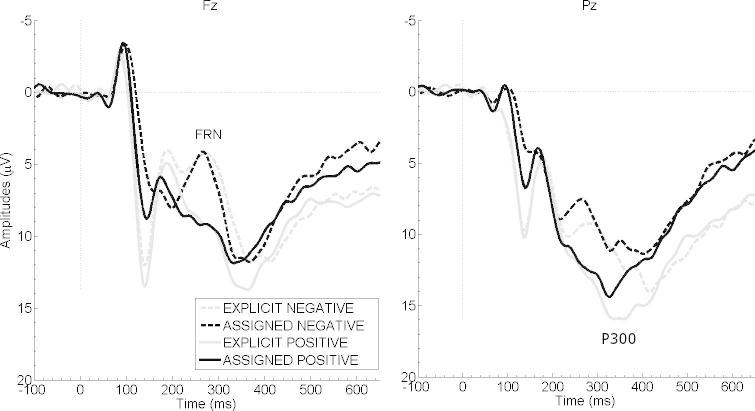
Grand average ERPs of experiment 2. Grand averages of the four conditions at electrode sites Fz (left) and Pz (right). Negative values are plotted upwards. Feedback presentation started at 0 ms, and lasted for 1000 ms.

**Fig. 3:**
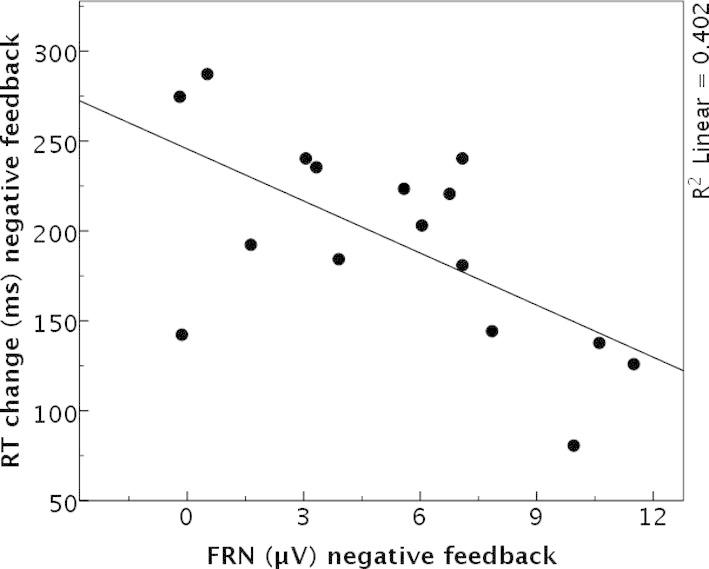
Scatter plot including a regression line of the FRN mean amplitudes (in μV) for negative feedback and the change in reaction times (in ms) after negative feedback of experiment 2.

Fig. 2 displays FRN and P3b amplitude courses of the four conditions of experiment 2. Analysis of FRN mean amplitudes revealed a main effect for *valence* (*F*(1,15) = 29.55, *p* < 0.001, $\eta_{p}^{2}$ = 0.66) with larger FRN amplitudes after negative than positive feedback. The factor *feedback type* had no impact on FRN amplitudes (*F*(1,15) = 0.16, *p* = 0.694), no interaction effect emerged either (*F*(1,15) = 0.04, *p* = 0.846). A significant correlation emerged between the trial-to-trial changes in response time after negative feedback and FRN amplitude values after negative feedback (*r* = −0.589, *p* = 0.016, *n* = 16), indicating that larger trial-to-trial adjustments were associated with more negative-going FRN amplitudes (Fig. 3). This relation was not observable after positive feedback (*p* > 0.556).

Analysis of P3b mean amplitudes yielded significant main effects for *feedback type* (*F*(1,15) = 26.18, *p* < 0.001, $\eta_{p}^{2}$ = 0.64) with larger (more positive) P3b amplitudes for explicit than for assigned feedback, and for *valence* (*F*(1,15) = 5.34, *p* = 0.036, $\eta_{p}^{2}$ = 0.26) with larger P3b amplitudes for positive than for negative feedback. No interaction effect emerged (*F*(1,15) = 0.15, *p* = 0.703). No significant correlations emerged between the trial-to-trial changes in response time and P3b amplitudes, although P3b amplitudes tended to be smaller after larger trial-to-trial changes after positive feedback (*r* = −0.494, *p* = 0.052).

## 4 Comparison of Experiments 1 and 2

For a direct comparison, we added the between-subject factor *experiment* to the applied ANOVA model *feedback type* × *valence* for the dependent variables trial-to-trial changes in response time, adjustments in response time, FRN mean amplitudes at Fz, and P3b mean amplitudes at Pz.

For the behavioral data, the factor *experiment* nearly reached significance for the number of correct adjustments (*p* = 0.056). More correct adjustments occurred in Experiment 2. No other significant effects emerged for the factor *experiment* (all p-values > 0.389) for trial-to-trial adjustments and number of correct adjustments.

For FRN mean amplitudes, a significant main effect of *valence* occurred (*F*(1,32) = 52.06, *p* < 0.001, $\eta_{p}^{2}$ = 0.62), with larger FRN amplitudes after negative than positive feedback. Factors *feedback type* (*F*(1,32) = 2.53, *p* = 0.122) and *experiment* (*F*(1,32) = 0.23, *p* = 0.632) were not significant. A significant *experiment* × *feedback type* (*F*(1,32) = 4.41, *p* = 0.044, $\eta_{p}^{2}$ = 0.12) interaction emerged. Tukey post hoc tests indicated a trend for larger FRN amplitudes after assigned than explicit feedback stimuli in experiment 1 (*p* = 0.052), but not in experiment 2 (*p* = 0.985). The remaining interactions were not significant (all *p*-values >0.466).

For P3b mean amplitudes, the factors *experiment* (*F*(1,32) = 8.59, *p* = 0.006, $\eta_{p}^{2}$ = 0.21), *feedback type* (*F*(1,32) = 38.47, *p* < 0.001, $\eta_{p}^{2}$ = 0.55), and *valence* (*F*(1,32) = 25.67, *p* < 0.001, $\eta_{p}^{2}$ = 0.45) were significant. P3b amplitudes were more positive in experiment 1 than 2. The interaction *feedback type* × *valence* reached significance (*F*(1,32) = 5.48, *p* = 0.026, $\eta_{p}^{2}$ = 0.15). Tukey post hoc tests indicated larger P3b amplitudes for positive than negative feedback for assigned (*p* < 0.001) and explicit feedback stimuli (*p* < 0.001); and larger P3b amplitudes for explicit than assigned feedback for positive (*p* < 0.001) and negative feedback stimuli (*p* < 0.001). Largest P3b amplitudes were observed after *explicit positive* feedback (all p-values < 0.001). The interactions *experiment* × *feedback type* (*F*(1,32) = 2.54, *p* = 0.121) and *experiment* × *valence* (*F*(1,32) = 2.57, *p* = 0.112) were not significant. The three-way interaction approached significance (*F*(1,32) = 3.34, *p* = 0.077).

### 4.1 Discussion

Blocking the presentation of explicit and assigned feedback yielded different results than experiment 1. Feedback type did not affect FRN amplitudes, which as in experiment 1 were more pronounced after negative compared to positive feedback. In contrast, P3b amplitudes were more pronounced after positive than negative feedback, and after explicit compared to assigned feedback. However, in contrast to experiment 1, no interaction between feedback type and valence was observed. Additionally, FRN amplitudes after negative feedback were related to behavioral response time adjustments.

These results therefore indicate that FRN (with trend-like significance) as well as P3b amplitude variation is influenced by the presentation mode. We hypothesized that blocking the two different feedback types led to more robust results since FRN effect sizes for feedback valence increased from experiment 1 to experiment 2 (from 0.59 to 0.66, respectively), a significant relation between FRN amplitude values and behavioral measures was observable only in experiment 2, and the direct comparison between the two experiments showed effects of feedback type only for the mixed presentation.

However, the observed differences between experiments 1 and 2 could be also caused by subtle individual differences between the participants of the two experiments, or other factors that had not been assessed in this between-subjects comparison. Therefore, to take into account the impact of individual differences and procedural differences between experiments, a third experiment was conducted in which the same participants in a within-subject design received explicit and assigned feedback stimuli in blocked and mixed presentation modes.

## 5 Experiment 3

The goal of this experiment was to replicate the results gained from the between-subjects comparison of experiments 1 and 2 using a full-factorial within-subject comparison. The task comprised of the same time estimation paradigm used in the previous experiments and explicit and assigned feedback stimuli were presented both randomly mixed and block-wise. Again, new participants were recruited.

### 5.1 Methods

#### 5.1.1 Participants

Initially, 24 volunteers (twelve females) participated in our third experiment. Two participants had to be excluded from further analysis due to technical problems with data acquisition. The remaining 22 participants (eleven females) were aged between 22 and 43 years, with a mean age of 27 ± 4.69 years. Participants were right-handed as assessed with the Edinburgh Handedness Inventory (Oldfield, 1971), had normal or corrected-to-normal vision, and reported no past or present neurological or psychiatric disorder. Again, age and gender distribution was comparable to the two other experiments. All participants gave written informed consent prior to the experiment. The same ethical standards applied as in experiments 1 and 2.

#### 5.1.2 Task

Experimental procedures were a combination of experiments 1 and 2. Half of the participants started with 200 trials presenting explicit and assigned feedback stimuli randomly mixed (mixed presentation); then they switched to 200 trials with blocks of the same feedback type (blocked presentation). Again, each block consisted of 50 trials where only symbols or faces indicated time estimation accuracy. The other half of the participants started with the blocked presentation (200 trials) and switched then to the mixed presentation (200 trials). In total, the experiment consisted of 400 trials. For the blocked presentation, it was randomly determined whether the first block for each participant consisted of explicit or assigned feedback stimuli. Thus, participants always knew which feedback type to expect after the first experimental trial.

#### 5.1.3 EEG data acquisition

Data acquisition and preprocessing procedures were nearly identical to experiments 1 and 2 apart from the following changes. EEG data were recorded from 61 Ag/AgCl ring electrodes with a DC amplifier set-up (NeuroPrax, neuroConn GmbH, Ilmenau, Germany) and sampled at 500 Hz for digital storage. Offline, EEG data were down-sampled to 250 Hz, high-pass filtered with a cut-off frequency of 0.1 Hz, and re-referenced to linked mastoids.

#### 5.1.4 Data analysis

Behavioral and EEG data analysis was identical to experiments 1 and 2. ICA was applied to remove residual eye movement-related activity (Delorme et al., 2007). Subject- and condition-wise averages were calculated for the eight conditions *blocked explicit negative*, *blocked explicit positive*, *blocked assigned negative*, *blocked assigned positive*, *mixed explicit negative*, *mixed explicit positive*, *mixed assigned negative*, and *mixed assigned positive*. Subsequently, FRN and P3b mean amplitudes were extracted 200–300 ms and 300–500 ms after feedback onset, respectively. All dependent variables were subjected to the same repeated-measures ANOVA model with the within-subject factors *presentation* (blocked, mixed), *feedback type* (explicit, assigned), and *valence* (negative, positive).

### 5.2 Results

#### 5.2.1 Behavioral results

The eight feedback conditions were evenly distributed across participants (blocked explicit negative: 12.7%, blocked explicit positive: 12.3%, blocked assigned negative: 13.0%, blocked assigned positive: 12.0%, mixed explicit negative: 12.8%, mixed explicit positive: 12.2%, mixed assigned negative: 12.8%, and mixed assigned positive: 12.2%). Consequently, participants received negative feedback in 51.3% of the 400 trials. In general, participants overestimated the one-second interval (mean time estimation response time was 1039 ms ±123). Concerning trial-to-trial changes in time estimation, a main effect of valence was observed (*F*(1,21) = 42.13, *p* < 0.001, $\eta_{p}^{2}$ = 0.67). Trial-wise reaction time adjustments were larger following negative than positive feedback. No significant effects were observed for the factors presentation (*F*(1,21) = 0.04, *p* = 0.848) and feedback type (*F*(1,21) = 0.13, *p* = 0.718) and all interaction terms (all *p*’s > 0.442). Participants adequately adjusted their time estimations (towards 1000 ms) in 52.4% of the trials after negative and in 44.2% after positive feedback. Concerning these correct adjustments, again only a main effect of valence emerged (*F*(1,21) = 24.71, *p* < 0.001, $\eta_{p}^{2}$ = 0.54), demonstrating more accurate time estimations following negative feedback. No significant effects were observed for the factors presentation (*F*(1,21) = 1.47, *p* = 0.239) and feedback type (*F*(1,21) = 1.45, *p* = 0.241) or any interaction terms (all *p*’s > 0.361).

Trial-to-trial changes in response time and number of correct adjustments are depicted in Table 5, mean FRN and P3b amplitudes in Table 6.

**Table 5:** Mean trial-to-trial change in response time and number of correct adjustments and corresponding standard deviations (SD) of experiment 3.

	Trial-to-trial change in response time		Number of correct adjustments	
	M	SD	M	SD
*Mixed condition*				
Explicit negative	178.83	73.07	27.68	5.69
Assigned negative	181.49	67.27	26.32	7.11
Explicit positive	139.88	52.65	22.95	6.27
Assigned positive	135.36	39.22	22.05	6.58

*Blocked condition*				
Explicit negative	180.28	60.38	25.09	8.19
Assigned negative	186.85	59.77	25.73	7.47
Explicit positive	135.69	44.19	22.14	6.34
Assigned positive	138.53	48.64	21.32	7.44

**Table 6:** Mean amplitude values and corresponding standard deviations (SD) of FRN at Fz and P300 at Pz in experiment 3 (*n* = 22).

		Explicit negative	Assigned negative	Explicit positive	Assigned positive
		*Mixed condition*			
FRN (Fz)	Mean amplitudes	1.26	2.23	4.28	4.43
	SD	7.30	5.90	8.34	6.17
P300 (Pz)	Mean amplitudes	11.07	8.66	14.51	11.77
	SD	7.01	5.31	7.18	5.92

		*Blocked condition*			
FRN (Fz)	Mean amplitudes	−0.09	1.24	3.96	5.22
	SD	6.94	5.30	7.18	5.61
P300 (Pz)	Mean amplitudes	9.68	7.04	12.18	9.16
	SD	6.63	4.89	6.53	5.15

#### 5.2.2 EEG results

**Fig. 4:**
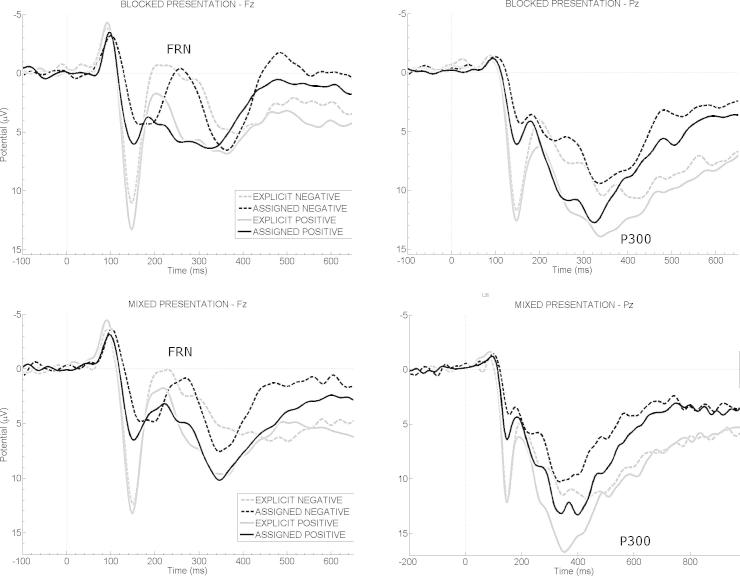
Grand average ERPs of experiment 3. Grand averages of the four blocked conditions at electrode sites Fz (left) and Pz (right) on the upper panel; the four mixed conditions at electrode sitzes Fz (left) and Pz (right) are depicted on the lower panel. Negative values are plotted upwards. Feedback presentation started at 0 ms, and lasted for 1000 ms.

**Fig. 5:**
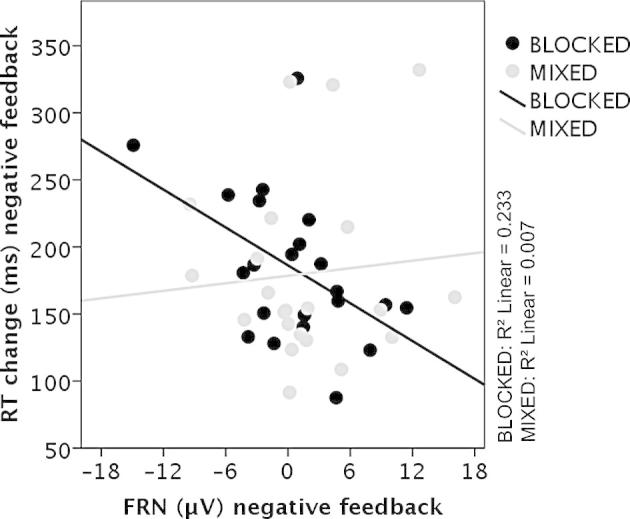
Scatter plot including regression lines of the FRN mean amplitudes (in μV) for negative blocked (black) and mixed (grey) feedback and the change in reaction times (in ms) after negative feedback of experiment 3.

Fig. 4 displays FRN and P3b amplitude courses of the eight conditions of experiment 3. Analysis of FRN mean amplitudes revealed a significant main effect of *valence* (*F*(1,21) = 47.99, *p* < 0.001, $\eta_{p}^{2}$ = 0.70) with larger (more negative) FRN amplitudes for negative than positive feedback. The main factors *presentation* (*F*(1,21) = 0.43, *p* = 0.518) and *feedback type* (*F*(1,21) = 1.20, *p* = 0.286) were not significant. Furthermore, a significant *presentation* × *valence* interaction emerged (*F*(1,21) = 8.03, *p* = 0.010, $\eta_{p}^{2}$ = 0.28). Tukey post hoc tests showed that FRN amplitudes were more pronounced after blocked compared to mixed presentation for negative feedback (*p* = 0.017) but not for positive feedback (*p* = 0.903). The remaining interaction effects were not significant (all *p*’s > 0.336). A significant correlation emerged between trial-to-trial changes in response times and mean FRN amplitudes for blocked negative feedback conditions (*r* = −0.483, *p* = 0.023) (Fig. 5). Again, larger trial-to-trial adjustments were associated with more pronounced FRN amplitudes. No such correlation emerged for the mixed presentation of negative feedback (*p* = 0.704) or the blocked (*p* = 0.112) or mixed presentation of positive feedback (*p* = 0.335).

Analysis of P3b mean amplitudes yielded significant main effects of *presentation* (*F*(1,21) = 7.78, *p* = 0.011, $\eta_{p}^{2}$ = 0.27), *feedback type* (*F*(1,21) = 10.01, *p* = 0.005, $\eta_{p}^{2}$ = 0.32), and *valence* (*F*(1,21) = 60.12, *p* < 0.001, $\eta_{p}^{2}$ = 0.74). P3b mean amplitudes were more positive after explicit than assigned feedback conditions. Moreover, a significant *presentation* × *valence* interaction occurred (*F*(1,21) = 7.22, *p* = 0.014, $\eta_{p}^{2}$ = 0.26). Tukey post hoc tests indicated that mixed positive feedback elicited the largest P3b amplitudes (all *p*’s < 0.001) and blocked negative feedback the smallest ones (all *p*’s < 0.024). No further significant interaction effect occurred (all *p*’s > 0.412). A significant correlation emerged between trial-to-trial changes in response time and mean P3b amplitudes for blocked negative feedback (*r* = −0.423, *p* = 0.050). Larger trial-to-trial adjustments were associated with less pronounced P3b amplitudes. No such correlation emerged for the mixed presentation of negative feedback (*p* = 0.339) or the blocked (*p* = 0.154) or mixed presentation of positive feedback (*p* = 0.507).

### 5.3 Discussion

Applying a within-subject design to investigate the effects of presentation mode and feedback type on FRN amplitudes yielded corroborating results in line with the two previous experiments. No generalized effect of feedback type was observable for FRN amplitudes. However, the blocked presentation led to most pronounced FRN amplitudes after negative feedback whereas no differentiation was observable between blocked and mixed presentation for positive feedback. Moreover, behavioral adjustments in reaction times were only associated to FRNs after negative feedback during the blocked presentation. Concerning P3b amplitudes, the effect of feedback type was again observed as well as largest amplitude deflections after mixed explicit feedback stimuli. Moreover, behavioral adjustments were also associated solely with the blocked feedback stimulus presentation.

## 6 General discussion

Our findings indicate that the type of feedback stimuli (i.e., emotional facial expressions versus abstract symbols) only modulated P3b but not FRN amplitudes in the current experiments. This contradicts our initial hypothesis of larger FRN amplitudes when presenting more salient explicit feedback stimuli. Although FRN amplitude variation has been reported in response to heightened stimulus saliency or heightened subjective motivational significance (Gehring and Willoughby, 2002, Yeung et al., 2005) of the respective stimulus, the way in which this salient outcome is delivered had no impact on FRN amplitude size per se in the current study. Miltner et al. (1997) reported no FRN amplitude variation in response to administering feedback via different modalities; the authors investigated visual, acoustic, and haptic feedback and reported no differences between the three. The present results suggest that also within the same sensory modality no FRN amplitude modulation is observable, even when adding inherent saliency to the feedback stimuli. Of note, a recent study reported FRN enhancement after manipulating saliency levels of feedback stimuli by adding monetary incentives in a block design (van den Berg et al., 2011). This experimental manipulation can be regarded as context manipulation which impacted ERP amplitudes. The physical appearance of the feedback stimuli might not have affected ERP amplitudes per se. Nevertheless, the present results indicate that the comparison of different FRN studies applying varying feedback stimuli within a similar experimental context yields comparable and valid results. Moreover, the FRN latency range might be too early to provide more than a quick and coarse evaluation of a stimulus as favorable/unfavorable (Hajcak et al., 2006, Pedroni et al., 2011), or as expected/unexpected (Hajcak et al., 2007, Pfabigan et al., 2011b). Thus, motivational or affective factors might only impact FRN amplitudes when being trait characteristics or when being presented prior to the actual feedback presentation, but not by an affective manipulation by the feedback stimulus itself. In line with this assumption, there is further evidence for long-term effects of negative affect on FRN amplitudes from clinical samples consisting of patients with anxiety and affective disorders (Olvet and Hajcak, 2008). However, future research is needed to corroborate the current findings because one has to be cautious when interpreting null-findings.

It has to be noted that the stimuli used for the assigned feedback conditions (+ and −) are highly overlearned symbols. Stimuli for which the assignment of correct and incorrect feedback has to be learned prior to the experiment might be even better suited to investigate the current research topic. A recent study applied a similar time estimation task using faces and meaningless symbols (x and o) as feedback stimuli in a block design (Schulreich et al., 2013). Indeed, the authors reported more negative FRN amplitudes for explicit than assigned feedback stimuli. However, their experimental timing and FRN assessment were slightly different than in the current study which could have also caused the observed differences between feedback stimuli in the study of Schulreich et al. (2013).

In contrast, P3b amplitudes were sensitive to the emotional content of the presented feedback. Admittedly, the P3b latency range is associated with more elaborate stimulus processing and evaluation (Polich, 2007). Thus, the finding of larger P3b amplitudes for positive as well as for explicit stimuli is in line with recent research (Bellebaum et al., 2010, Pfabigan et al., 2011b). Indeed, Nieuwenhuis et al. (2005) proposed that the P3b amplitude variation might reflect the extent to which processed information is subjectively or motivationally salient. More precisely, the authors assumed that P3b amplitude variation on the scalp reflects the activity of the locus coeruleus-norepinephrine system which is involved in classifying salient and non-salient events. Furthermore, the present results might also fit into the independent coding model proposed by Yeung and Sanfey (2004). The authors assumed that feedback valence and feedback magnitude might be processed separately in the brain. According to Yeung and Sanfey (2004), FRN amplitude variation reflects outcome valence, whereas P3b amplitude variation reflects the magnitude of the respective outcome. Concerning the present data, one could argue that explicit positive feedback, i.e. the smiling face, was perceived as the most rewarding stimulus of the present experiment in comparison to the explicit negative and the assigned feedback stimuli. Indeed, O’Doherty et al. (2003) demonstrated that smiling compared to neutral facial stimuli led to enhanced activation in brain areas highly associated with reward processing. However, larger P3b amplitudes after explicit than assigned feedback stimuli could also be explained by stimulus complexity. More complex stimuli require more attentional resources which are reflected in larger P3b amplitudes (Isreal et al., 1980, Johnson, 1986). Unfortunately, the current study is not suitable to answer this question. Future studies should address the topic of stimulus complexity and feedback processing in detail.

Furthermore, our three experiments addressed the question whether the presentation mode impacts FRN and P3b amplitudes. To our knowledge, this is the first EEG study demonstrating that the presentation mode of feedback types – randomly mixed (experiment 1) versus blocked (experiment 2) versus a within-subject design with mixed and blocked presentation – can have a direct impact on ERPs related to feedback processing. Differences in the two presentation modes were reflected in more obvious FRN peaks in the grand average ERPs, higher FRN effect sizes, and stronger correlations between FRN and behavioral performance measures when a blocked presentation mode was used. The direct comparison of both experiments as well as the within-subject design (experiment 3) further emphasized the advantages of the blocked presentation mode. Only a few studies addressed the research question whether different presentation modes impact ERP amplitudes. Wilson et al. (1998) observed decreases in P300 amplitudes elicited by a warning tone when their participants adapted to a constant level of difficulty during blocked compared to random stimulus presentation. The authors assumed that the observed P300 decrease was related to the fact that the warning tone delivered no relevant information concerning task difficulty any more during the blocked presentation. Another study investigating ERPs related to emotional stimulus content observed no effect of presentation mode (presenting similarly valenced stimuli blocked or intermixed) on late positive potential (LPP) amplitude variation (Pastor et al., 2008). The LPP is another positive-going ERP observed about 400–700 ms after stimulus onset reflecting motivated attention during emotional picture processing (Bradley et al., 2007).

To summarize, presentation mode has to be taken into consideration when planning to use different types of feedback stimuli within the same experiment.

## 7 Conclusions

In summary, we conducted three experiments to address the question whether additional saliency of explicit feedback stimuli modulates FRN and P3b amplitudes compared to feedback stimuli with assigned valence information which were assumed to be less salient. P3b, but not FRN amplitudes, were prone to our saliency manipulation which might be explainable via stimulus evaluation processes assessing motivational stimulus significance reflected in P3b amplitudes or stimulus complexity. Additionally, our results suggest that applying a block-design when using different types of feedback stimuli within the same experiment is advisable. Our findings might also be relevant for the assessment and treatment of patients with mental disorders since feedback processing plays an essential part during learning processes which are often targeted by therapeutic interventions. Additionally, the FRN component is often used as measure in studies with clinical populations (Groen et al., 2008, Mies et al., 2011, Morris et al., 2011, O’Toole et al., 2012). Therefore, our findings are also relevant for researchers who conduct such clinical studies.
